# One-Step Robot-Assisted Complete Urinary Tract Extirpation in Man with End-Stage Renal Disease on Dialysis: The First Case Report

**DOI:** 10.3390/curroncol30050385

**Published:** 2023-05-16

**Authors:** Che-Hsueh Yang, Chao-Yu Hsu, Yi-Sheng Lin, Min-Che Tung, Yen-Chuan Ou

**Affiliations:** Division of Urology, Department of Surgery, Tungs’ Taichung MetroHarbor Hospital, Taichung 435, Taiwan; b101098093@tmu.edu.tw (C.-H.Y.); t12197@ms.sltung.com.tw (Y.-S.L.); t1142@ms.sltung.com.tw (M.-C.T.)

**Keywords:** carcinoma, transitional cell/surgery, perioperative care, robotics, renal dialysis/methods, treatment outcome, urologic neoplasms/surgery, urologic surgical procedures/methods, case reports

## Abstract

**Simple Summary:**

If patients with end-stage renal disease under dialysis suffered from urothelial carcinoma, extirpating the whole urothelium of urinary tracts in one-stage surgery could offer benefits of removing highly suspicious recurrent cancers in the future in advance and simplifying the bothersome monitoring strategies. Postoperative infection could be lowered if dialysis was carried out via autogenous vascular fistula, rather than temporary catheters. However, it was possible that autogenous vascular fistula became dysfunctional and required surgical revisions. For patients after extirpating the whole urinary tracts, kidney transplants with ileal conduits were feasible to re-establish the filtration system of the bodies. However, this choice was reserved for those without lymph nodes involvement or metastasis.

**Abstract:**

Urothelial carcinoma (UC) could be observed in urinary bladder (UBUC) and upper urinary tracts (UTUC). In the National Comprehensive Cancer Network guidelines for bladder cancer, extirpative surgery is indicated in certain cases. However, some extreme cases might also need the extirpation of the majority of the urinary tract, which is called complete urinary tract extirpation (CUTE). We present a patient diagnosed with high-grade UBUC and UTUC. He underwent dialysis for end-stage renal disease (ESRD) at the same time. Considering his non-functional kidneys and removing his high-risk urothelium at the same time, we performed robot-assisted CUTE to extirpate both his upper urinary tracts, urinary bladder, and prostate. In our experience, the console time was not significantly elongated, and the perioperative course was uneventful. To our knowledge, this is the first case report adopting a robotic system in such an extreme case. We conclude that robot-assisted CUTE is worth further study regarding its oncological survival outcomes and perioperative safety in patients with ESRD on dialysis.

## 1. Introduction

Urothelial carcinoma (UC) in the genitourinary system mostly occurs in the urinary bladder (UBUC) and upper tracts (UTUC). Approximately 90% of UC is UBUC and 10% is UTUC. From the above description, UBUC and UTUC seem only to be different in their locations at the urinary tracts. However, there is more and more evidence of a remarkable disparity between them, such as epidemiology, pathogenesis, biomarkers, and prognosis after treatment [1,2]. In epidemiology, synchronous UBUC and UTUC is rarely reported, which is estimated to be around 2% in the literature [3]. Indications of extirpative surgery for UTUC include multifocal tumors, flat or sessile tumors, tumors at the mid and proximal ureters with technical challenges, a size over 1.5 cm, high-grade tumors, or muscle-invasive tumors [4]. In UBUC, extirpative surgery is indicated in patients with non-muscle-invasive tumors with very high risk features, Bacillus Calmette–Guérin unresponsiveness or intolerance, or muscle-invasive UBUC [4]. However, in special conditions, such as end-stage renal disease (ESRD), many considerations should be taken into account. In epidemiology, UBUC was found to be highly related to ESRD in both women and men [5]. Aside from this, in patients with ESRD, the gains of certain chromosomal loci in DNA repair genes contributed to high-stage and high-grade UTUC [6]. In this way, the tumor characteristics in those with ESRD might be different from those without ESRD. In recurrent or muscle-invasive UBUC on stable dialysis, radical cystectomy with concurrent nephroureterectomy might have potential survival benefits. The 5-year overall survival rate could be increased by around 30% [7]. Because the detection and identification of synchronous or metachronous UC in patients with ESRD on dialysis were difficult [8], extirpating suspicious malignant urothelium as much as possible in one-step surgery could be considered in patients without mortality risk factors, such as tumors combined with infection or unstable hemodynamics [7,9]. The common high mortality risk factors in these groups undergoing additional nephroureterectomy included complications related to dialysis and infection [7].

One-stage or staged complete urinary tract extirpation (CUTE) could be considered as another choice for patients with special conditions, such as staged surgery for UC with frequent recurrences and one-stage surgery for UC with a potentially high recurrence risk [10,11]. In patients with ESRD on dialysis, this surgery could eradicate potentially recurrent UC and might provide better survival benefits. However, this surgery might also be accompanied by high morbidities and mortalities, and patients on dialysis for ESRD are highly vulnerable. During the operation, special considerations in patients with ESRD include coagulation, body fluid balance, electrolyte balance, and changes in cardiovascular functions [12]. Currently, CUTE was only reported in small-sized studies [9,13], and tailoring the perioperative care for patients on dialysis for ERSD after CUTE was rarely mentioned. Moreover, robot-assisted CUTE was reported in only a few case reports [14,15] and was not studied as often as open or laparoscopic CUTE. Thus, we report our first experience and outcome of adopting robot-assisted CUTE in men with ERSD on dialysis.

## 2. Case

### 2.1. Initial Assessment

**Figure 1:**
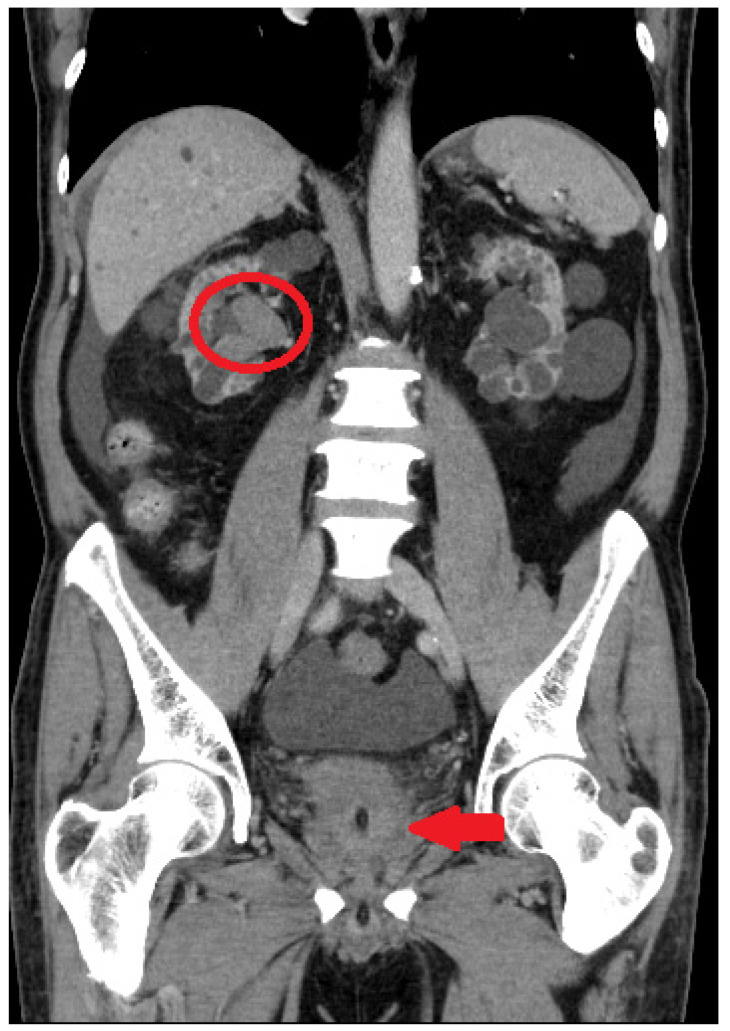
Initial assessment with computed tomography. Dense blood clot (red arrow) and right hydronephrosis (red circle) were observed.

**Figure 2:**
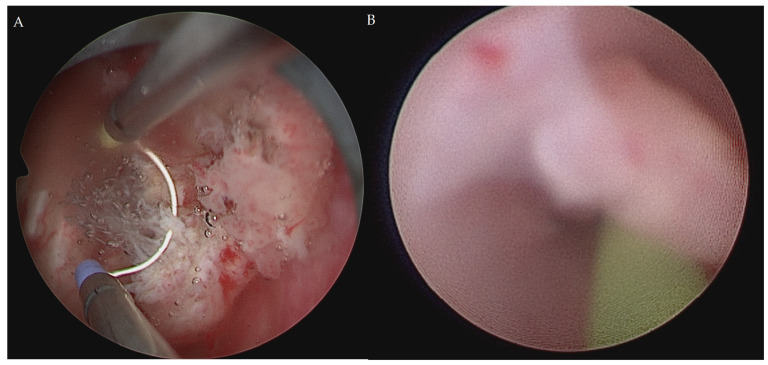
Cystoscopy for draining blood clots. After draining blood clots, TURBT was performed to collect the specimens for microscopic observation (**A**). Another tumor at the right ureteropelvic junction was noted under ureteroscopy (**B**).

A 60-year-old male hepatitis B virus carrier had undergone peritoneal dialysis (PD) for 7 years for ESRD due to chronic glomerulonephritis. He first visited our clinics for gross hematuria and acute urine retention. After computed tomography (CT), a dense blood clot and right hydronephrosis were observed (Figure 1). Since the blood clot was too dense to be evacuated via urethral catheter, cystoscopy was further arranged. After draining all blood clots, two tumors in the urinary bladder (UB) were identified. The specimens were sent for microscopic examination after performing transurethral resection of the bladder tumor (TURBT) (Figure 2A). A right ureteroscopy was also performed to rule out any obstruction causing hydronephrosis, and a tumor was noted under ureteroscopy. The biopsy was performed to retrieve the specimen for microscopic observation (Figure 2B). After the operation, a right ureteral stent was indwelled in the right ureter. The pathological results demonstrated that there were high-grade T1 papillary UBUC and UTUC at the right ureteropelvic junction.

### 2.2. Robot-Assisted CUTE

The patient’s initial serum creatinine was 14.48 mg/dL and estimated glomerular filtration rate was 3.7 mL/min/1.73 m^2^. The first vascular fistula was established between the left radial artery and cephalic vein at 2 weeks after the TURBT and ureteroscopy. At 2 months after fistula establishment, the robot-assisted CUTE was scheduled. Hemodialysis (HD) was carried out one day before robot-assisted CUTE.

**Figure 3:**
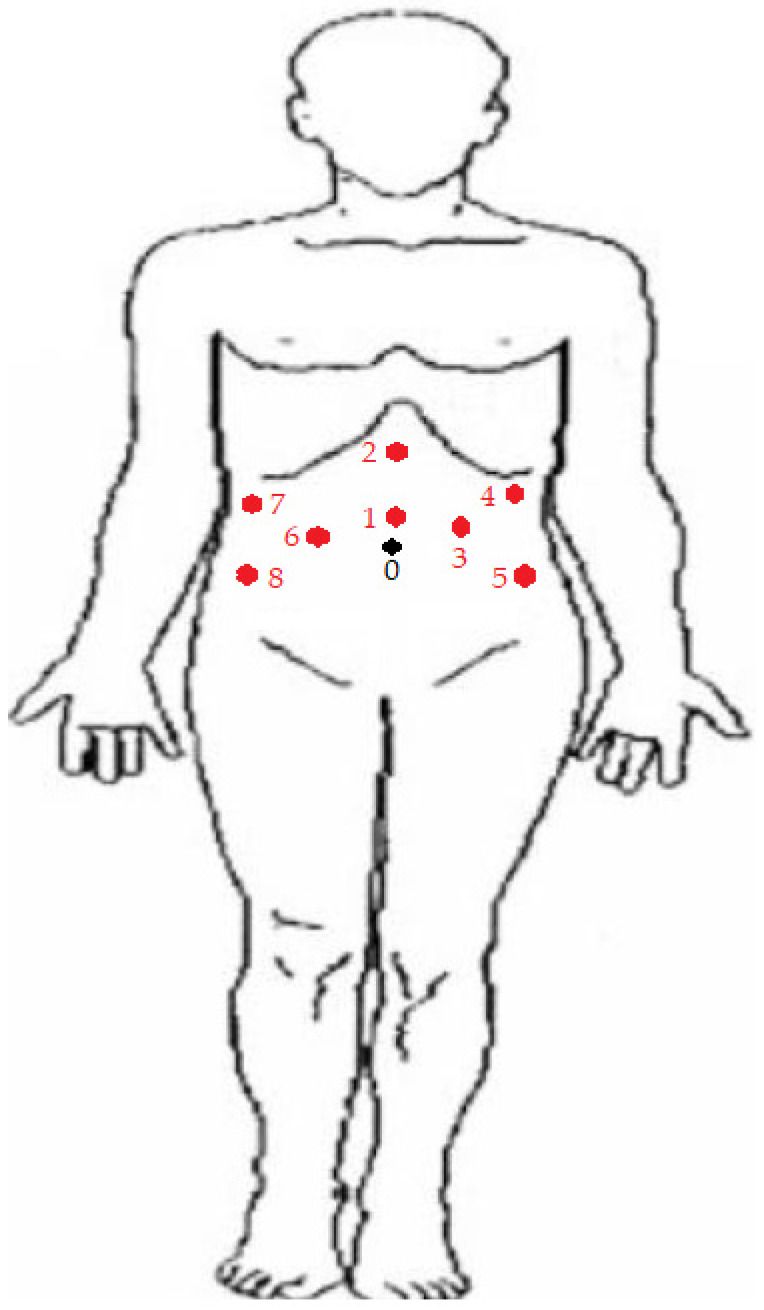
Port placement of robot-assisted CUTE. Port 0 was marked as the site of umbilicus. During the left nephrectomy, ports 1 to 5 were adopted. Ports 1 and 2 were 12 mm and 5 mm in size, respectively. They functioned as assistant ports during the surgery. A camera port was placed at No. 3. The robotic right and left arms were docked at ports 5 and 4, respectively. All ports from No. 3 to 5 were 8 mm in size. During the right nephrectomy, ports 1 and 2 were adopted for the same role as the assistant ports. The camera was placed at port 6. The right and left arms were placed at ports 7 and 8, respectively. Ports from No. 6 to 8 were all 8 mm in size. During the cystectomy and prostatectomy, ports 1, 3, 5, 6, 7, and 8 were adopted. The camera was placed at port 1, and the right arm was placed at port 6. Two left arms were docked at ports 3 and 5. Ports 7 and 8 were used as 5 mm and 12 mm assistant trocars, respectively. All ports except Nos. 7 and 8 were 8 mm in size.

**Figure 4:**
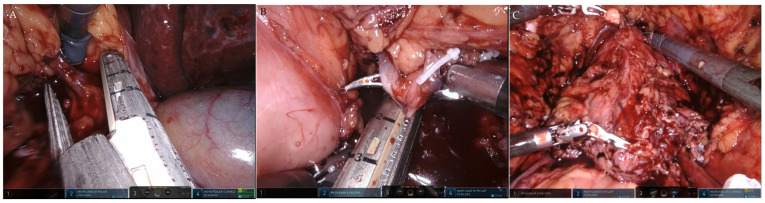
One-step robot-assisted CUTE. The operation was carried out in the order of (**A**) right nephroureterectomy, (**B**) left nephroureterectomy, and (**C**) cystoprostatectomy.

The robot-assisted CUTE was carried out using the da Vinci Surgical Xi System (Intuitive Surgical, Inc., Sunnyvale, CA, USA). The port placement is illustrated in Figure 3. The device was undocked 2 times to change positions, once for the right lateral decubitus and again for the 25-degree Trendelenburg position. The operation was carried out in the order of right nephroureterectomy, left nephroureterectomy, and cystoprostatectomy (Figure 4). The adrenal gland on the left side was preserved, while the right side was removed during the surgery. The console time was 384 min, and total blood loss was 1650 mL. A total of 250 mL of blood was transfused immediately after the operation. Two drainage tubes were placed in the bilateral flanks after the operation, and another urethral catheter was inserted for drainage. There were no intraoperative complications.

**Figure 5:**
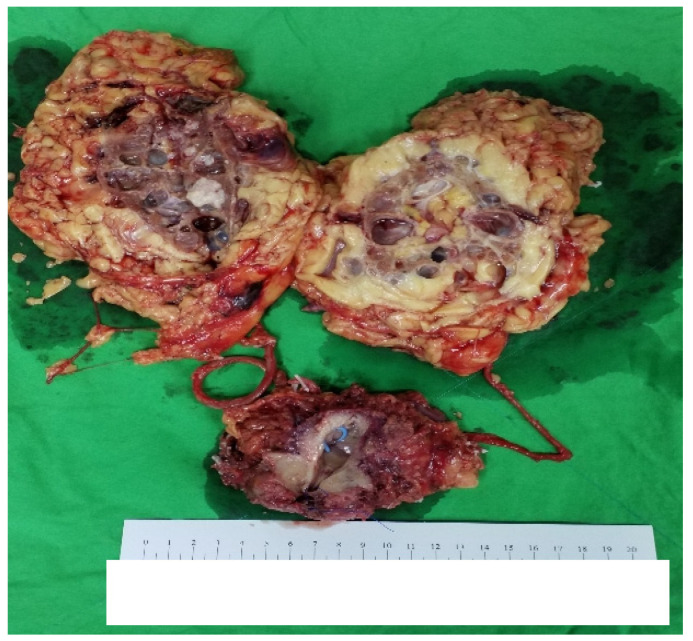
The general appearance of the specimen.

Grossly (Figure 5), there was 1 nodular lesion (1 × 1 cm) situated in the UB, and the mucosa of the UB was totally embedded. In addition, 1 tumor (5 × 4 cm) was present in the right renal pelvis. The bilateral kidneys showed multiple cystic lesions, and the largest size was measured as 3 × 2 cm.

**Figure 6:**
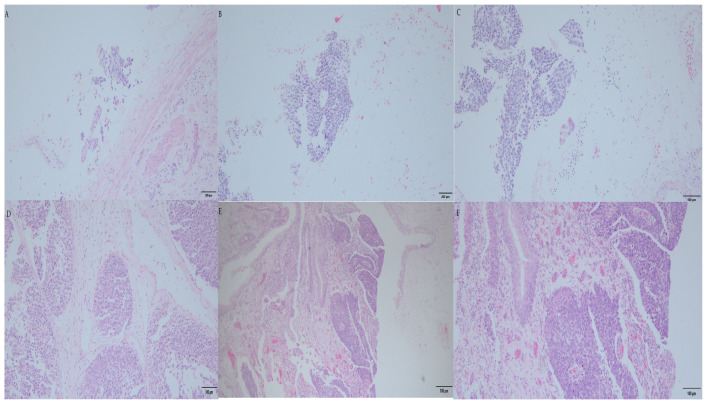
The microscopic pathology of the surgical specimen. (**A**) Right noninvasive high-grade UTUC at upper ureter (magnified by 200×). (**B**) Infiltrating high-grade UTUC was noted at the right renal pelvis, and the peripelvic fat was invaded (magnified by 200×). (**C**) Infiltrating high-grade UTUC was examined the right renal pelvis and the peripelvic fat (magnified by 400×). (**D**) This high-grade UTUC at the right renal pelvis was observed with the neoplastic cells showing a solid growth pattern, large plasmacytoid cells and pleomorphic nuclei with scanty cytoplasm, and frequent mitosis with invasion to the fatty tissue. Lymphovascular invasion was not found (magnified by 400×). (**E**) Infiltrating high-grade UBUC was observed with invasion to lamina propria (magnified by 100×). (**F**) Infiltrating high-grade UBUC was examined (magnified by 200×).

Microscopically (Figure 6), there was infiltrating high-grade UBUC with invasion to the lamina propria. At the right kidney pelvis, infiltrating high-grade UTUC was noted with invasion to the peripelvic fat. The tumor showed high-grade UC with the neoplastic cells in a solid growth pattern, large plasmacytoid cells, pleomorphic nuclei with scanty cytoplasm, and frequent mitosis with invasion to the fatty tissue. Lymphovascular invasion was not found. At the right upper ureter, another noninvasive high-grade UTUC was observed. At the left kidney and ureter, only chronic pyelonephritis was noted, and no tumors were seen at the left upper urinary tract. There were no tumors noted at the prostate, prostatic urethra, seminal vesicle, or bilateral vas deferens. There were 12 lymph nodes dissected from the right renal hilar, paracaval, precaval, and retrocaval regions. All of them were negative. There were no templates for lymph node dissection at the left upper urinary tract since preoperative malignancies were not proved. No regional lymph nodes were examined in the specimen of the left urinary tract.

### 2.3. Postoperative Care and Follow-Up

The total hospital stay was 13 days. The HD was resumed on the first day after surgery and was scheduled once every two days. The left drainage tube and urethral catheter were removed on the fourth day after the operation, and the right one was removed on the eleventh day after the operation. After removing the right drainage tube, discharge with periodic follow-up was arranged.

## 3. Discussion

The first documented report of CUTE can be traced back to 1992 [10], performed with a 3-step approach. This case was initially a noninvasive UBUC, refractory to intravesical instillation, and recurred after cystectomy, nephroureterectomy, and partial nephrectomy. After systemic chemotherapy, the rest of the kidney was extirpated, and the patient lived on HD. Later, the largest case series was published [11]. CUTE was then introduced as one of the therapeutic choices in multifocal urinary tract UC and various special conditions, such as ESRD under dialysis. Patients with ESRD, status under dialysis or status post kidney transplant were generally considered to be immunocompromised [16], and they contributed to the high incidence and recurrence rate of urinary tract UC [17,18]. Furthermore, patients with non-functional kidneys requiring urinary diversion after operation were difficult to survey for recurrence after operation [19]. In those patients with non-functional kidneys and a high risk of recurrence, aggressive and preemptive initial treatment would possibly yield a better recurrence-free survival.

Theoretically, one-step CUTE might be suitable for those with ESRD [10]. Especially in the Asian population, the relation between UC and ESRD was stronger than in other ethnicities [10,20]. For example, the incidence of UC in ESRD was 0.9% in England [21], but the incidence rate was 1.2% in Taiwan [22]. Based on standardized incidence ratios, renal cancer was more prevalent than UC in Western society [20], while UC was more prevalent in Asian ethnicities [22]. However, there was only one comparative study comparing one-step CUTE and multi-step CUTE [23]. The study found no improvements in 5-year overall, recurrence-free, and cancer-specific-free survival. Moreover, the authors opposed one-stage CUTE, since the mortality was only observed in the one-stage CUTE group. However, the recruitment numbers were disproportionate and underpowered in the case and control groups, leading to an overt inflation in the estimation of regression analysis. Thus, their argument for the inferiority of one-stage CUTE is questionable, and this topic should be further elucidated by the upcoming comparative case–control studies. We should keep in mind that ESRD itself would already be the negative prognostic indicator even with one-step CUTE [12]. ESRD patients with an age over 66 years, a Charlson comorbidity index (CCI) > 2, and invasive UBUC or UTUC should be informed of inferior prognosis in advance of CUTE [13].

In the perioperative period, major complications (Clavien–Dindo > 2) occurred in around 30–40% of patients undergoing CUTE [9,13,23], which elongated hospital stays by around 1 week [9]. Although ESRD and dialysis should be given special consideration and were associated with worse prognosis, they were not necessarily related to major postoperative complications. Those with an age over 62 years and CCI ≥ 5 were vulnerable to major complications [9]. The most common major complication was arteriovenous (AV) fistula dysfunction [9,23], estimated to occur in 8–10% of patients undergoing CUTE. In our case, we established the AV fistula at 2 months before CUTE. In the choice of access, an autogenous fistula would be better than a postoperative temporary catheter in terms of mortality and infection [24]. Although AV fistula dysfunction was the most common major complication requiring surgery, our experience demonstrated that it was a feasible method avoiding mortality and infection after CUTE. Generally, an AV fistula would require a median time of 47 days to maturation [24]. Thus, patients with PD could be clinically assessed for this preoperative preparation if they were at a high risk of having a worse prognosis. However, the literature does not explain the post-CUTE AV fistula dysfunction and its associated factors, and thus the risk still needs to be communicated to patients when they consider the pre-CUTE AV fistula establishment.

Regarding the surgery method, open and laparoscopic are the two major methods in the literature. Robot-assisted CUTE was first reported around three years ago [14], and there are still no large cohort studies. In the first case report, the operative time was similar to ours, but there was no blood transfusion after surgery [14] (Table 1). In our experience, robot-assisted CUTE only required more effort in changing the positions but did not elongate the total operative time [9]. In robot-assisted simultaneous nephroureterectomy and cystectomy, the major complication rate was low [25], indicating that adopting the robot-assisted method in CUTE might resolve the high major postoperative complication rate. However, the oncological outcomes of robot-assisted CUTE are still lacking due to the limited data in the literature. Once the oncological outcomes are determined, patients with ESRD on dialysis may further benefit from a transplant with an ileal conduit after removing the majority of the urothelium suspected of malignancies and confirming non-recurrence for a certain period of surveillance [14]. Although there is still no consensus on the period between the localized tumor extirpation and transplant, we should keep in mind that the positive lymph nodes should be considered as one of the contraindications. Since patients had positive lymph nodes in previous malignancies, their 5-year disease-free survival would only range from 0% to 32% [26].

## 4. Conclusions

Robot-assisted CUTE was safe and feasible in patients with simultaneous UBUC and UTUC and with ESRD on dialysis. However, there are still no large-scale cohort studies discussing this. Thus, studies on oncological survival outcomes are worth conducting.

## Figures and Tables

**Table 1:** The published literature of robot-assisted CUTE.

	Case	Operative Time	Blood Loss	Postoperative Morbidity or Mortality	Tumor and Pathological Stage
Carrion et al. [14]	65-year-old male with multifocal low-grade UBUC and recurrences Newly diagnosed UTUC, UBUC, and UC at urethra	360 min	460 mL; no blood transfusion Hemodialysis was started at the 6th day after surgery	Glans infection (Clavien–Dindo grade 2)	Right renal pelvis: pT3N0Mx Left renal pelvis: high-grade pT1N0Mx Urinary bladder: low-grade pTa Prostatic–bulbar–membranous–penile urethra: low-grade pTa
Yee et al. [15]	61-year-old female with previous history of renal transplant Muscle-invasive UBUC Robot-assisted cystectomy and nephrectomy of the native kidneys Ileal conduit was created	543 min	500 mL; no blood transfusion	No	Urinary bladder: high-grade pT1N0
This case	60-year-old male with ESRD and on dialysis for 7 years Preoperative biopsy of T1 high-grade UBUC and UTUC at right ureter	384 min	Blood loss of 1650 mL; had blood transfusion Hemodialysis was started the next day after surgery	No	Right renal pelvis: pT3N0Mx Right upper ureter: high-grade pT1N0Mx Urinary bladder: high-grade pT1NxMx

## Data Availability

The data supporting this case report are all within this article.
